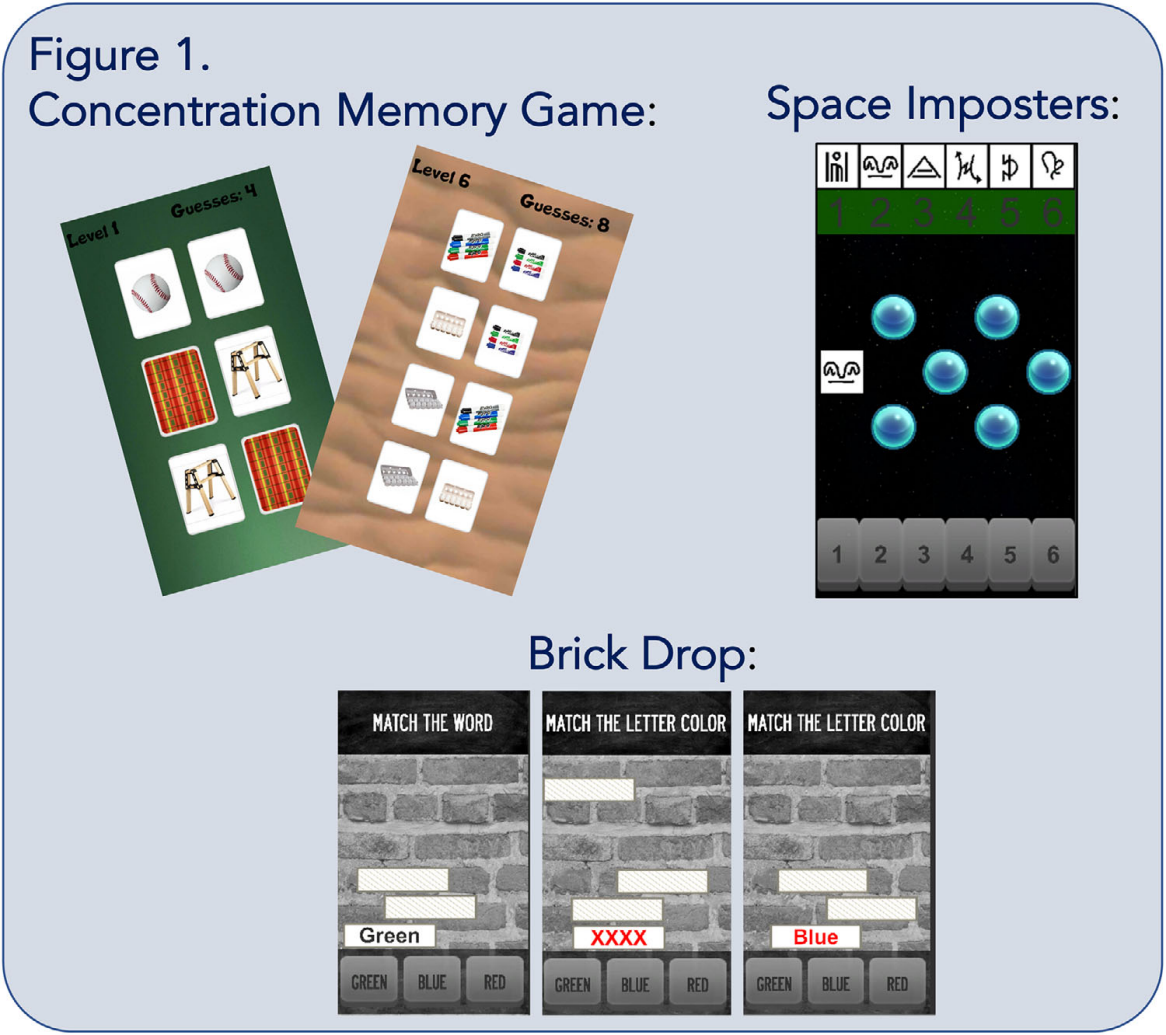# Remote App‐Based Cognitive Assessment in Aging and Pre‐Clinical Alzheimer’s Disease in a Diverse Sample of Older Adults

**DOI:** 10.1002/alz.094357

**Published:** 2025-01-09

**Authors:** Dawn Mechanic‐Hamilton, Valerie Humphreys, Alex Prusky, Kimberly Halberstadter, David A Wolk

**Affiliations:** ^1^ Penn Alzheimer’s Disease Research Center, University of Pennsylvania, Philadelphia, PA USA; ^2^ University of Pennsylvania, Philadelphia, PA USA

## Abstract

**Background:**

Mobile, valid and engaging cognitive assessments are essential for detecting and tracking change in research participants and patients at risk for Alzheimer’s Disease and Related Dementias (ADRDs). This pilot study aims to determine the feasibility and generalizability of an at‐home, app‐based cognitive assessment, the mobile cognitive app performance platform (mCAPP), to detect cognitive changes associated with aging and preclinical AD.

**Method:**

mCAPP includes three gamified tasks (Figure 1): (1) a “concentration” memory task that includes learning and matching hidden card pairs with increasing memory load, pattern separation features (lure vs. non‐lure), and spatial memory (2) a stroop‐like task (“brick drop”) with speeded word and color identification and response inhibition components and (3) a digit‐symbol coding‐like task (“space imposters”) with increasing pairs. Participants used mCAPP at home for two weeks and completed the NACC UDS3 and additional paper and pencil tests. Participants included ninety‐three adults (72% female; age = 72.5±5.5, education = 16.4±2.6; 49.5% White, 49.5% Black/African American, 1% Multiracial) without cognitive impairment enrolled in the Penn ADRC cohort. A subgroup also completed longitudinal mCAPP testing and amyloid PET imaging.

**Result:**

Participants played 11±4 sessions over two weeks, with 61% playing more than the assigned sessions. Most participants (97%) used a smartphone. Usability rating was 6.3±0.8 (1‐7 scale). Most participants rated the difficulty of the tasks as “just right” for Concentration (93%), Brick Drop (84%), and Space Imposters (87%) after playing the games for the first time. 70% reported preferring mobile device cognitive assessment to standard in‐person cognitive batteries. All tasks showed lower performance with increasing cognitive load (p’s<.05). mCAPP measures correlated with paper and pencil tests from analogous domains on paper and pencil measures (p’s<.01). Participants with a positive amyloid PET scan showed a trend toward slower and less accurate performance on some mCAPP measures.

**Conclusion:**

This pilot study shows mCAPP usability for at‐home use in a diverse cohort of older adults. Performance across measures indicate initial reliability and validity of mCAPP. Future work will include the evaluation of mCAPP performance relationships with biomarkers and longitudinal data analysis. Remote, accessible, and reliable cognitive assessment has the potential to reduce barriers to research participation and clinical care.